# Increasing Use of a Postpartum and Newborn Chatbot among Birthing Individuals and Caregivers: Development and Implementation Study

**DOI:** 10.2196/81844

**Published:** 2026-01-09

**Authors:** Jessica N Rivera Rivera, Marjanna Smith, Shrey Mathur, Katarina E AuBuchon, Angela D Thomas, Hannah Arem

**Affiliations:** 1 Healthcare Delivery Research, MedStar Health Research Institute, 100 Irving St. NW, Washington, DC, 20010, United States, 1 4436921138; 2 Center for Biostatistics, Informatics and Data Science, MedStar Health Research Institute, Washington, DC, United States; 3 Palliative Care Research Group, MedStar Health Research Institute, Washington, DC, United States; 4 Department of Oncology, Georgetown University, Washington, DC, United States

**Keywords:** postpartum period, newborn care, health information, chatbot, implementation

## Abstract

**Background:**

The 42 days following childbirth are a high-risk period for birthing individuals and newborns. We created 2 rule-based chatbots, 1 for birthing individuals and 1 for newborn caregivers, to deliver information on postpartum and newborn warning signs, follow-up care, and other relevant resources during this high-risk period.

**Objective:**

This study aims to examine strategies for implementing the chatbot following discharge from a large hospital center, initial chatbot reach, and subsequent reach after chatbot refinement based on end-user feedback.

**Methods:**

Reach was defined as the number of users opening the chatbot out of those who received it. Birthing individuals’ demographic (age, ethnicity, race, language, and insurance type) and clinical characteristics (delivery method and prenatal care location) and newborns’ time in the hospital were obtained from the medical record. Descriptive statistics, chi-square tests, and multiple logistic regression models were used to analyze the association between demographic and clinical characteristics and chatbot reach.

**Results:**

Both chatbots were developed and revised based on clinician, community, and patient feedback. Overall, 65.9% (4933/7489) of newborn caregivers discharged between October 2, 2022, and January 15, 2025, opened the newborn chatbot, and 63.6% (4140/6505) of birthing individuals discharged between November 21, 2022, and January 15, 2025, opened the postpartum chatbot. Older age (odds ratio [OR] 1.02, 95% CI 1.01-1.03), Black race (OR 0.73, 95% CI 0.61- 0.88; reference: White), languages other than English or Spanish (OR 1.90, 95% CI 1.21-2.98; reference: English), receipt of prenatal care external to the hospital system (federally qualified health center: OR 0.52, 95% CI 0.45-0.60; Kaiser: OR 0.34, 95% CI 0.29-0.39; reference: within the hospital system), and public insurance (OR 0.72, 95% CI 0.64-0.82; reference: private insurance) were significant predictors of postpartum chatbot reach. Older age (OR 1.02, 95% CI 1.01-1.03), Black race (OR 0.61, 95% CI 0.50-0.74; reference: White), receipt of prenatal care external to the hospital system (federally qualified health center: OR 0.50, 95% CI 0.44-0.57; Kaiser: OR 0.30, 95% CI 0.26-0.35; reference: within the hospital system), public insurance (OR 0.63, 95% CI 0.55-0.71) and self-pay (OR 0.56, 95% CI 0.38-0.83; reference: private insurance), and newborn time in the hospital of 2‐4 days (OR 1.21, 95% CI 1.09-1.35; reference: less than 2 d) were significant predictors of newborn chatbot reach. Including a Spanish-language version in the newborn chatbot improved reach among Spanish-preferring caregivers (from 58% to 66.2%), but additional chatbot content revision and the addition of chatbot information to discharge paperwork did not change chatbot reach.

**Conclusions:**

While there were differences in chatbot reach by patient demographics, the chatbot showed delivery of time-sensitive information and support to >60% of individuals. This intervention demonstrated that chatbots can be used to supplement patient care and help bridge the gaps in connecting patients to care and support after hospital discharge. Future work should address additional ways to improve chatbot reach and explore the impact on targeted health outcomes.

## Introduction

The 42-day period after childbirth is widely viewed as high-risk both for the birthing individual and for the newborn [1-3]. Access to timely information about potential warning signs for when to seek emergency care, along with proper follow-up care and support, has the potential to improve maternal and infant health outcomes after discharge from the hospital. Top causes of death for birthing individuals in the 42-day period after delivery are mental health conditions, cardiovascular conditions, and infections [4], while top causes of mortality among infants are birth defects, preterm birth and low birth weight, and sudden infant death syndrome [5]. Previous work has cited the importance of monitoring women post-delivery, with an estimated 15% of severe maternal morbidity cases occurring after discharge [6]. Furthermore, a prior randomized control trial found that enhanced caregiver education via SMS text messaging, timed to the infant’s age and most common reasons for emergency department visits, reduced emergency department visits in infants’ first year of life [7]. In recent years, SMS text messaging services such as Text4Baby (National Healthy Mothers, Healthy Babies Coalition and Voxiva) [8,9] and apps such as BabyScripts (1EQ, Inc) [10,11] have been developed to provide information to patients during the prenatal and postpartum periods on maternal and infant health.

Chatbots are another mobile health (mHealth) strategy used to deliver timely information. Maternal chatbots are an acceptable and feasible strategy for postpartum and caregiving information and support [12,13]. For example, Rosie is a chatbot that leverages artificial intelligence (AI) to deliver personalized assistance related to pregnancy, labor, postpartum care, and newborn care [13,14]. Another chatbot, Dr. Joy, is an obstetric and mental health-related question-and-answer knowledge-based chatbot that also leverages AI for prenatal and postpartum care [15]. While these chatbots are effective in disseminating relevant health information among select patients, they have been deployed in small trials, as opposed to part of standard of care.

We developed and piloted 2 rule-based chatbots (one for birthing individuals and one for newborn caregivers) to provide timely health information and resources, including connection to care. This study aims to describe the process of developing and piloting an mHealth program aimed at improving education on postpartum and newborn warning signs and appropriate connection to care and resources after hospital discharge. We also aim to describe the chatbot reach (number of patients who opened the chatbot out of those who received it) among patients discharged from a large mid-Atlantic hospital that serves a socioeconomically and racially diverse population overall. We assessed reach after initial launch and after refining the chatbot in response to patient feedback. We hypothesized that with each chatbot refinement, there would be an improvement in reach. Finally, we explored how individuals’ demographic and clinical factors were associated with reach for each chatbot.

## Methods

### Overview

This study was prepared in accordance with the iCHECK-DH (Guidelines and Checklist for the Reporting on Digital Health Implementations) [16].

### Chatbot Development, Initial Implementation Strategies, and Setting

The postpartum and newborn chatbots were developed as part of a larger initiative, Safe Babies Safe Moms (SBSM), which was aimed at reducing infant and maternal disparities in the District of Columbia (DC) [17]. These chatbots were designed with key goals of helping patients connect with care teams (eg, information about recommended pediatric and postpartum appointments, list of pediatricians in the area), educating them on warning signs, and providing additional postpartum and newborn information and resources (eg, breastfeeding and wound healing after C-section) [18]. The chatbots operated on a rule-based system with fixed logic for interaction (ie, patients could not ask open-ended questions). Content was developed by a multidisciplinary team of experts in obstetrics, pediatrics, social work, psychiatry, mHealth, and health equity, designed to meet the needs of diverse patients.

We created 2 separate chatbots to account for instances where a birthing individual and newborn did not go home together (eg, adoption, surrogacy, neonatal intensive care unit, or postpartum complication) and provide appropriate information at the appropriate time. Thus, within a nonprofit health care system, caregivers of newborn patients discharged at a large, mid-Atlantic hospital between August 29, 2022, and January 15, 2025, received the newborn chatbot, and birthing individuals discharged between November 21, 2022, and January 15, 2025, received the postpartum chatbot within 24 hours of hospital discharge as standard of care. Individuals discharged after January 15, 2025, did not receive the chatbots due to the funding period ending.

**Figure 1.**
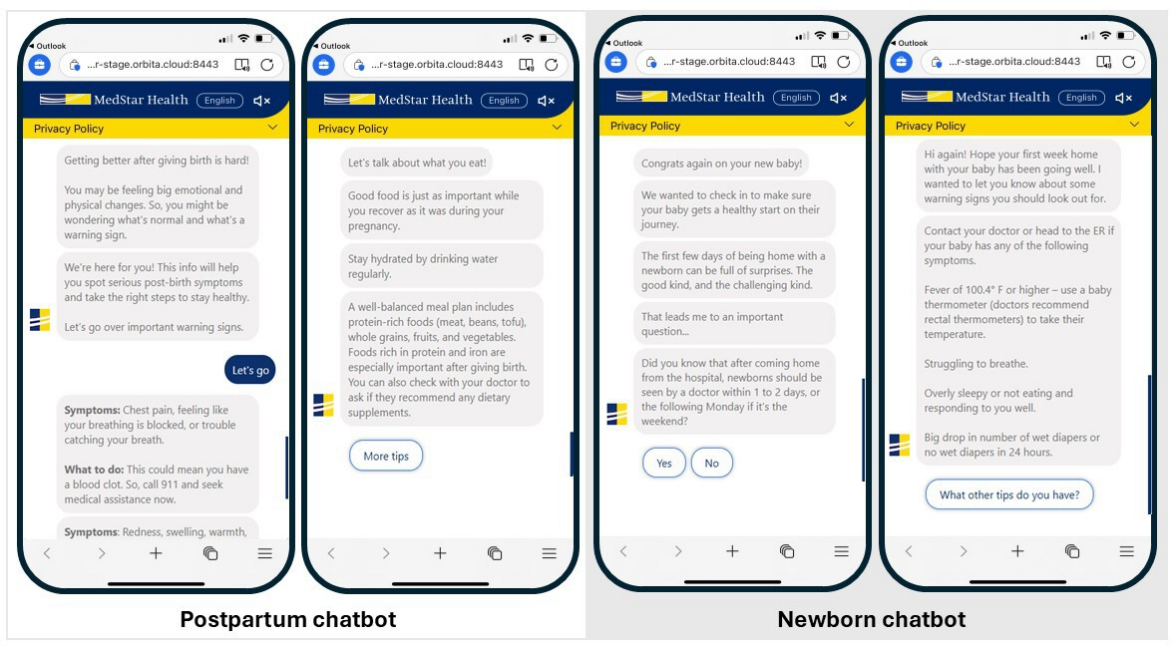
Chatbot screenshots of the postpartum and newborn chatbots.

The postpartum chatbot messages were sent in the morning, delivering messages weekly for the first 42 days post-discharge (Figure 1 and Multimedia Appendix 1). The newborn messages started 24 hours after discharge, asked about whether a newborn visit was scheduled, offered support and resources when the caregiver answered no, and followed up with weekly informational outreach messages. A total of 7 messages with unique content were sent for the postpartum chatbot, and 5 messages were sent for the newborn chatbot. Each unique set of chatbot content is referred to as an experience. The topics included in each chatbot experience are listed in Table 1. In short, topics included appointment reminders, warning signs, nutrition recommendations, and developmental milestones for newborns and postpartum care. Both postpartum and newborn messages were sent as standard of care to all individuals who delivered at the hospital, except for one community clinic that opted to only implement the newborn chatbot because they already had a postpartum follow-up process and did not want to confuse patients. There was some content tailoring in the postpartum chatbot; for instance, birthing individuals who had a C-section received information about wound care.

**Table 1.** Newborn and postpartum chatbot reach for caregivers and birthing individuals, and topics covered by each chatbot experience.

Experiences and topics covered in the chatbot^a^	General reach by experience, n/N (%)
Newborn chatbot experience^b^	
Experience 1 (day 1 post discharge)	
Original: pediatric appointment reminder; resources to address common challenges for scheduling an appointment (only offered to patients without an appointment); parental leave	1757/4438 (39.6)
Revised: pediatric appointment reminder; resources to address common challenges for scheduling an appointment (offered to all patients); parental leave	1285/2935 (43.8)
Experience 2 (day 7)	
Original: pediatric warning signs; newborn sleep recommendations	930/4384 (21.2)
Revised: pediatric warning signs; newborn sleep recommendations (added recommendations for premature newborns)	652/2989 (21.8)
Experience 3 (day 14)	
Original: newborn nutrition recommendations; resources for breastfeeding and formula; food assistance program	973/4320 (22.5)
Revised: newborn nutrition recommendations; resources for breastfeeding and formula; colic education; newborn bowel movement education; food assistance program	691/3053 (22.6)
Experience 4 (day 28)	
Original: newborn developmental milestones	1104/4192 (26.3)
Revised: pediatric 1-month visit; tips for communicating with providers; newborn developmental milestones; recommended activities with newborns	886/3181 (27.9)
Experience 5 (day 38)	
Original: recommended pediatric visits and vaccines	1608/4104 (39.2)
Revised: 2-month checkup and recommended vaccines; support groups; resources for diapers and baby essentials	1126/3269 (34.4)
Postpartum chatbot experience^c^	
Experience 1 (day 1 post discharge)	
Original: postbirth warning signs; baby blues	1336/3895 (34.3)
Revised: postbirth warning signs; baby blues and recommendations	907/2610 (34.8)
Experience 2 (day 3)	
Original: postbirth warning signs; postpartum check-up; recovering after a C-section; resources to address common challenges for scheduling a postpartum check-up	1317/3874 (34)
Revised: postbirth warning signs; postpartum recovery; recovering and management after a C-section or vaginal birth; breastfeeding tips and resources; pain management; paid family leave	846/2631 (32.2)
Experience 3 (day 7)	
Original: nutrition	903/3846 (23.5)
Revised: nutrition and resources; stress management tips, postpartum check-up and resources to address common challenges for scheduling or attending a postpartum check-up	544/2659 (20.5)
Experience 4 (day 14)	
Original: postpartum depression; sleep recommendations	691/3794 (18.2)
Revised: postpartum depression; sleep recommendations and tips	390/2711 (14.4)
Experience 5 (day 21)	
Original: family planning and sex after birth	1002/3728 (26.9)
Revised: family planning and sex after birth	706/2777 (25.4)
Experience 6 (day 28)	
Original: pelvic floor - Kegel exercises	656/3660 (17.9)
Revised: physical activity; Pelvic floor - Kegel exercises	511/2845 (18)
Experience 7 (day 42)	
Original: social support; postpartum depression	413/3560 (11.6)
Revised: social support and resources; resources for postpartum depression; health care after birth	250/2945 (8.5)

a The topics covered in each experience were revised, and the new content for all experiences was launched on February 21, 2024, for both chatbots. Thus, the total number of patients who received the new content will differ by experience.

b Caregivers of newborns discharged from the hospital between October 2, 2022, and January 15, 2025, received the newborn chatbot. A total of 116 caregivers who received the newborn chatbot were excluded due to missing data by experience.

c Only birthing individuals discharged from the hospital between November 21, 2022, and January 15, 2025 received the postpartum chatbot.

### Electronic Health Record Data Extraction and Integration

The chatbot system relied on structured electronic health record (EHR) data to identify discharge dates for birthing individuals and newborns. A native EHR data extraction program using Cerner Command Language was designed to retrieve relevant patient information including contact information, preferred language, and prenatal care provider. The data extraction was fully automated and ran daily. These data were securely transmitted to the chatbot vendor system via the enterprise Interface Engine using secured File Transfer Protocol to ensure end-to-end transport layer security, consistent with industry-standard security practices. Upon receiving the contact list, the chatbot vendor delivered the chatbot content through SMS text messaging and email. The vendor generated daily analytics files that included user reach metrics (eg, overall chatbot use) that were securely transferred back to the health care system.

### Study Design and Participants

This is a pragmatic implementation study where we described the strategies used to develop the postpartum and newborn chatbots and performed a cross-sectional analysis to evaluate the postpartum and newborn reach. Because patients received the chatbots as standard of care, we included all the birthing individuals and caregivers of newborn patients discharged from a large, mid-Atlantic hospital in the data analyses. Newborn chatbot reach was evaluated for newborn patients discharged from October 2, 2022, to January 15, 2025, who received the newborn chatbot. We were unable to report chatbot delivery and reach during the first month of chatbot launch due to inconsistent documentation by the vendor; these issues were resolved during team meetings. For postpartum reach, all birthing individuals discharged from November 21, 2022, to January 15, 2025, and who received the postpartum chatbot were included in the analysis.

### Measures

#### Implementation Strategies and Chatbot Reach

The chatbots were designed to fill a gap in follow-up after patient discharge, particularly among those patients who did not seek care within the health care system before or after birth. Implementation strategies specific to increasing reach and engagement for the chatbot focused on developing stakeholder relationships, reexamining implementation, and engaging consumers. The primary outcome of this analysis is chatbot reach and is defined as the number of people who opened the chatbot link out of those who received outreach messaging. Chatbot reach data were obtained from the chatbot vendor, and the research team linked the data back to the patient’s medical record.

#### Demographic and Clinical Data Collection

Birthing individuals’ and newborns’ demographic and clinical characteristics were obtained from the EHR. These independent variables were categorized as follows: birthing individual age (<20, 20‐29, 30‐39, 40+ years), ethnicity (Hispanic or non-Hispanic), race (Black or African American, White, other, or unknown), preferred language (English, Spanish, other [eg, Amharic, Arabic, French, etc], or unknown), and insurance type (private or commercial, public, other, self-pay, or unknown). Regarding medical history, we included delivery method (vaginal birth or C-section) for the birthing individuals, newborns’ weight at birth (very low birthweight: <1500g, low birthweight: 1500g to <2500g, or normal birthweight: ≥2500g), gestational age (<37 wk or ≥37 wk), time in the hospital between birth and discharge (<2 d, 2‐4 d, or >4 d), and prenatal care location (care within the MedStar Health system where they delivered, Kaiser [where both care and insurance are provided within the same system], other external clinics (largely federally qualified health centers [FQHCs] or unknown sources of care). Birthweight and gestational age data were only available for newborns discharged from October 2, 2022, to September 30, 2024, as they were obtained from the EHR for a prior SBSM project [17].

### Data Analysis

We used descriptive statistics to describe reach and chi-square tests to evaluate demographic and clinical factors related to the postpartum and newborn chatbot reach. We also assessed changes in reach after modifying chatbot outreach and content. We used backward selection of the independent variables (birthing individual age, ethnicity, race, preferred language, insurance type, and prenatal care location) to finalize the multivariate logistic regression models. For the postpartum chatbot reach, we also included birthing individual delivery method (vaginal vs cesarian), and for the newborn chatbot, we included time in the hospital. In addition, we tested the impact of adding birthweight and gestational age as independent variables to the newborn chatbot logistic regression limiting the population to the date range October 2, 2022, to September 30, 2024, (as these 2 variables were only available between these dates).

### Ethical Considerations

The study protocol was approved by the Institutional Review Board (IRB) from MedStar Health Research Institute (IRB #5741). This project was completed in accordance with the ethical standards of the MedStar Health Research Institute IRB and the Helsinki Declaration of 1975 and the 2000 revision. We did not ask for consent or compensate patients in this study as the chatbots were automatically sent to patients as standard of care. Our IRB granted a Health Insurance Portability and Accountability Act waiver to link chatbot engagement to the patients’ demographic and clinical data (eg, age, race, preferred language, type of insurance, and prenatal care location). Identifiers were needed to link patients to their relevant EHR information. All patients’ identifiers were deleted from all files after data analysis was completed.

## Results

### Implementation Strategies

#### Developing Stakeholder Relationships

Before developing the chatbot, we met with the clinical leaders in labor and delivery, including physicians, nurses, and administrators. We also consulted with our community partners on key content and outreach strategies, as two-thirds of those delivering at the target hospital seek prenatal and postpartum care outside of the hospital’s health care system. We then identified a lead in each of the clinical areas involved (pediatrics, labor and delivery, and behavioral health) to help with the development of the chatbot content.

We iteratively developed the chatbot content, incorporating feedback from birthing individuals and caregivers (n=9) recruited from two pediatric clinics, who reviewed the content and participated in an individual semistructured interview. We notified providers and health care system leaders once the program was ready to launch and invited feedback on integration with existing workflows. Throughout this project, clinical leads were invited to participate in scientific abstracts to increase a sense of buy-in and were given opportunities to reflect on progress. Additionally, we presented ongoing successes and challenges to the parent project’s strategic advisory board, which includes clinical and community maternal and infant health equity experts, to solicit feedback. In response to provider feedback about the busy pace and competing priorities of the labor and delivery unit, we automated outreach to eliminate any need to ask providers to enroll patients. Despite the automation, building stakeholder relationships was vital to ensure that clinical teams were comfortable with their patients receiving the outreach, optimize the outreach content, and increase the likelihood that they would support introducing it into discharge paperwork.

#### Iterative Processes, Including Purposefully Re-Examining the Implementation and Reaching Consumers

**Figure 2.**
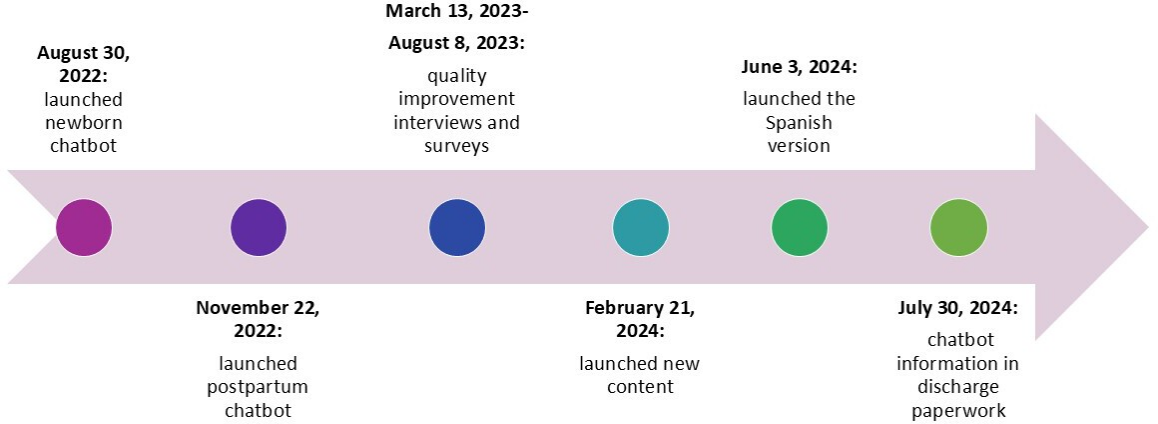
Postpartum and newborn chatbot implementation and refinement timeline.

The timeline for chatbot launch and iterative refinement is shown in Figure 2. The research team met with the chatbot vendor weekly to review data. With input from the research team, the vendor created a dashboard to monitor reach and certain engagement metrics (eg, user response to the question about scheduling their first newborn pediatric appointment). After assessing baseline rates of reach and preliminary engagement, between March and August of 2023, we collected qualitative and quantitative feedback from diverse users (46% identified as Black, 25% as Hispanic, and 54% had public insurance) to solicit input on increasing reach and use of chatbot content [18].

Based on initial reach data and patient feedback (reported elsewhere [18]), we created the following changes: (1) we modified the outreach messages to include more information about each week’s content, (2) we added relevant topics and resources within each chatbot experience (revised topic content listed in Table 1), (3) we translated the chatbot content into Spanish, (4) we added information about the chatbot to the discharge paperwork so that patients who reviewed the paperwork might have a frame of reference for the outreach.

The outreach messages were changed from a generic script to specify the topics covered in the corresponding experience. Overall, language was revised to improve readability and clarity (eg, “postpartum” was replaced with “after giving birth”). The chatbot content was changed in various ways (Table 1). First, we added additional tailored information for recovery after a C-section or vaginal birth, and options to read more about breastfeeding, with sensitivity to patient experiences around whether they chose to or were able to breastfeed. Second, additional resources such as Maternal Mental Health Hotline, Postpartum Support International, Breastfeeding Center of Greater Washington, and Safe Sleep Program from DC Department of Health were added. Third, we included a voice-over feature to facilitate reviewing the chatbot information using audio, as well as an option for patients to download the content as a PDF so that they could refer back to the information and share it with their partners or family members. We reviewed the revised content with 5 patients at an obstetrics clinic and 5 caregivers at a pediatric clinic to solicit in-person feedback via brief interviews. The interviewer took structured notes on patients’ and caregivers’ recommendations to share with the study team. Recommendations included: slowing down the speed that chatbot messages added text, shortening the messages, bolding the most important information, and adding a summary of the information included in the shared links. The speed at which new messages appeared in the chatbot was slowed to 2500 milliseconds, and we divided the information into more segments where patients could choose response options. This allowed us to shorten the messages and increase the potential for interaction within the chatbot. The updated outreach messages and chatbot content were launched on February 21, 2024.

A Spanish version of each chatbot was implemented on June 3, 2024, after professional translation, given that Spanish is the second most common primary language among our patient population. We also revised the outreach message to include a Spanish explanation for changing the chatbot language to Spanish as preferred language in the EHR may not capture all appropriate patients. Finally, on July 30, 2024, we added screenshots about the chatbot outreach and content in the discharge paperwork for both birthing individuals and newborns to inform patients about the chatbot and address concerns that the chatbot outreach messages were potentially a scam.

#### Chatbot Reach Overall

A total of 6505 individuals out of 6684 birthing individuals (97.3%) discharged from the hospital successfully received the postpartum chatbot outreach message; those who did not receive it either had a landline phone number or did not have a working phone number or email in the EHR. Overall, 7489 out of 7525 caregivers (99.5%) successfully received the chatbot outreach message for each newborn. Less than 1% of recipients opted out of the chatbots. Approximately 63.3% of patients had a valid email (postpartum: 4107/6505 and newborn: 4757/7489), and 99.9% (postpartum: 6505/6505 and newborn: 7488/7489) had a valid phone number. A total of 6107 birthing individuals/caregivers received the postpartum and newborn chatbot messages, 398 birthing individuals only received the postpartum chatbot, 1076 unique caregivers only received the newborn chatbot, and 298 caregivers received additional newborn chatbot messages for each newborn (due to multiparous, or births at different times; eg, 8 individuals had 3 newborns each during the project period).

Approximately two-thirds of recipients opened messages from either chatbot (Table 2). Newborn chatbot reach by experience ranged from 41.3% (3042/7373, experience 1) to 21.5% (1582/7373, experience 2), and the postpartum chatbot reach ranged from 34.5% (2243/6505, experience 1) to 10.2% (663/6505, experience 7; Table 1). Birthing individuals and caregivers who had a valid phone and email had a significantly higher chatbot reach (postpartum chatbot: 2980/4107, 72.6% and newborn chatbot: 3561/4756, 74.9%) than patients with only a valid phone number (postpartum chatbot: 1160/2398, 48.4% and newborn chatbot: 1372/2732, 50.2%). Only one caregiver had a valid email only and was not reached. For both chatbots, approximately 57% of users opened the messages by SMS text messaging only (postpartum: 2388/4140 and newborn: 2831/4933), 14% by email only (postpartum: 601/4140 and newborn: 678/4933), and 28% by both SMS text messaging and email (postpartum: 1151/4140 and newborn: 1424/4933).

**Table 2.** Patients’ demographic and clinical characteristics by postpartum and newborn chatbot reach.

Patients’ demographic	Postpartum chatbot^a^				Newborn chatbot^b^			
	Total received	Total reach	Reach^c^, %	*P* value^d^	Total received	Total reach	Reach^c^, %	*P* value^d^
Total	6505	4140	63.6		7489	4933	65.9	
Age (years)				<.001				<.001
<20	285	153	53.7		332	189	56.9	
20‐29	2310	1349	58.4		2746	1669	60.8	
30‐39	3466	2337	67.4		3945	2720	69.0	
40+	444	301	67.8		466	355	76.2	
Race				<.001				<.001
Black	2926	1749	59.8		3527	2217	62.9	
White	940	713	75.9		1001	823	82.2	
Other	1294	860	66.5		1468	953	64.9	
Unknown	1345	818	60.8		1493	940	63.0	
Ethnicity				.01				<.001
Hispanic	470	318	67.7		517	355	68.7	
Non-Hispanic	3937	2539	64.5		4589	3108	67.7	
Other	101	67	66.3		113	69	61.1	
Unknown	1997	1216	60.9		2270	1401	61.7	
Language				.003				<.001
English	5633	3589	63.7		6498	4321	66.5	
Spanish	699	436	62.4		793	480	60.5	
Other	113	86	76.1		123	93	75.6	
Unknown	60	29	48.3		75	39	52.0	
Insurance				<.001				<.001
Private	2670	1866	69.9		2955	2197	74.4	
Public	2941	1749	59.5		3498	2103	60.1	
Self-pay	106	67	63.2		120	72	60.0	
Other	79	59	74.7		68	52	76.5	
Unknown	709	399	56.3		848	509	60.0	
Delivery method				.13				N/A^e^
Vaginal	3987	2509	62.9		N/A	N/A	N/A	
Cesarean	2518	1631	64.8		N/A	N/A	N/A	
Baby weight^f^ ^g^				N/A				.07
Very low birthweight (<1500 g)	N/A	N/A	N/A		64	47	73.4	
Low birthweight (1500 to <2500 g)	N/A	N/A	N/A		633	396	62.6	
Normal birthweight (≥2500 g)	N/A	N/A	N/A		5863	3890	66.4	
Gestational age^f^ ^g^				N/A				.003
<37 weeks	N/A	N/A	N/A		738	452	61.3	
≥37 weeks	N/A	N/A	N/A		5822	3881	66.7	
Time in hospital after birth ^g^				N/A				<.001
<2 days	N/A	N/A	N/A		3070	1972	64.2	
2‐4 days	N/A	N/A	N/A		3304	2257	68.3	
>4 days	N/A	N/A	N/A		1115	704	63.1	
Prenatal care location				<.001				<.001
Within the hospital integrated health system	2895	2148	74.2		3042	2380	78.2	
Kaiser clinics^h^	1234	630	51.1		1342	721	53.7	
Other external clinics (eg, FQHCs)^i^	1864	1072	57.5		2609	1546	59.3	
Unknown clinics	512	290	56.6		496	286	57.7	

a Among birthing individuals discharged from the hospital between November 21, 2022, and January 15, 2025, who received the postpartum chatbot.

b Among caregivers of newborns discharged from the hospital between October 2, 2022, and January 15, 2025, who received the newborn chatbot.

c Reach is defined as the number of people who opened the chatbot link out of those who received outreach messaging.

d Chi-square statistics.

e N/A: not applicable.

f Data are only available through September 30, 2024.

g Data specific to newborn patients obtained from the electronic health record.

h Kaiser clinics are external clinics where both care and insurance are provided within the same system.

i FQHC: federally qualified health center.

Significant differences in reach were identified by age, ethnicity, race, preferred language, and insurance type across both chatbots (Table 2; all significant *P* values <.001). Birthing individuals who opened messages were more likely to be 30 years and older (2638/3910, 67.5%) compared to 29 years and younger (1502/2595, 57.9%), Hispanic (318/470, 67.7%) compared to non-Hispanic (2539/3937, 64.5%); White (713/940, 75.9%) compared to Black (1749/2926, 59.8%), had private insurance (1866/2670, 69.9%) compared to public insurance (1749/2941, 59.5%), and had prenatal care within the hospital’s integrated health system (2148/2895, 74.2%) compared to external prenatal clinics (Kaiser patients 630/1234, 51.1%), other largely FQHC clinics ( 1072/1864, 57.5%), unknown prenatal care location (290/512, 56.6%). No significant differences in reach were found by the delivery method (ie, vaginal vs C-section).

Similarly, newborn chatbot caregiver reach was higher among birthing individuals who were 30 years and older ( 3075/4411, 69.7%) compared to 29 years and younger (1858/3078, 60.4%), White (823/1001, 82.2%) compared to Black (2217/3527, 62.9%), and had private insurance (2197/2955, 74.4%) compared to public insurance (2103/3498, 60.1%). Newborn chatbot reach was also higher when the newborn had a gestational age of 37 weeks or more (3881/5822, 66.7%) compared to a gestational age of less than 37 weeks (452/738, 61.3%), stayed in the hospital 2‐4 days (2257/3304, 68.3%) compared to less than 2 days (1972/3070, 64.2%) and more than 4 days (704/1115, 63.1%), and had prenatal care within the hospital integrated health system (2380/3042, 78.2%) compared to external prenatal clinics Kaiser (721/1342, 53.7%), other largely FQHC clinics (1546/2609, 59.3%), and unknown prenatal care location (286/496, 57.7%). No significant differences were found for birth weight.

#### Postpartum Chatbot Analyses

In the final postpartum multivariate logistic regression model, we included age, race, preferred language, type of insurance, and prenatal location (Table 3). The odds of opening the postpartum chatbot were significantly lower for individuals identified in the EHR as Black (odds ratio [OR] 0.73, 95% CI 0.61- 0.88) compared to White individuals. Patients with a preferred language of “other” (OR 1.90, 95% CI 1.21-2.98) had greater odds of postpartum chatbot reach compared to English-preferring patients. Patients with public insurance (OR 0.72, 95% CI 0.64-0.82) and unknown insurance (OR 0.57, 95% CI 0.47-0.69) had lower odds of postpartum chatbot reach compared to individuals with private insurance. Patients who received prenatal care at clinics external to the hospital, including Kaiser clinics (OR 0.34, 95% CI 0.29-0.39), other clinics (OR 0.52, 95% CI 0.45-0.60), or unknown clinics (OR 0.45, 95% CI 0.37-0.55), had lower odds of postpartum chatbot reach compared to patients who received prenatal care within the hospital integrated health system. Age was also a significant predictor, with each one-year increase in age associated with a 2% higher likelihood of chatbot reach (OR 1.02, 95% CI 1.01-1.03).

**Table 3.** Multivariate logistic regression for postpartum chatbot reach among birthing individuals discharged from the hospital between November 21, 2022, and January 15, 2025. Reach is defined as the number of people who opened the chatbot link out of those who received outreach messaging.

Characteristics	OR^a^ (95% CI)		*P* value
Age (continuous)	1.02 (1.01-1.03)		<.001
Race			
White (reference)	N/A^b^		N/A
Black	0.73 (0.61-0.88)		<.001
Other	1.10 (0.88-1.37)		.40
Unknown	0.92 (0.75-1.13)		.40
Language			
English (reference)	N/A		N/A
Spanish	1.04 (0.86-1.27)		.66
Other	1.90 (1.21-2.98)		.005
Unknown	0.69 (0.41-1.16)		.16
Insurance			
Private (reference)	N/A		N/A
Public	0.72 (0.64-0.82)		<.001
Self-pay	0.75 (0.49-1.14)		.18
Other	1.07 (0.63-1.82)		.80
Unknown	0.57 (0.47-0.69)		<.001
Prenatal location			
Within the hospital system where delivery occurred (reference)	N/A		N/A
Kaiser clinics^c^	0.34 (0.29-0.39)		<.001
Other external clinics (eg, FQHCs)^d^	0.52 (0.45-0.60)		<.001
Unknown clinics	0.45 (0.37-0.55)		<.001

a OR: odds ratio.

b N/A: not applicable.

c Kaiser clinics are external clinics where both care and insurance are provided within the same system.

d FQHC: federally qualified health center.

#### Newborn Chatbot Analyses

For the final newborn multivariate logistic regression model, we included the birthing individual’s age, race, insurance type, prenatal care location, and their newborn’s time in the hospital (Table 4). Age was a significant predictor, with each one-year increase in age associated with a 2% higher likelihood of chatbot reach (OR 1.02, 95% CI 1.01-1.03). Birthing individuals who were listed in the EHR as Black (OR 0.61, 95% CI 0.50-0.74), other race (OR 0.73, 95% CI 0.59-0.91), and unknown race (OR 0.74, 95% CI 0.60-0.91) had lower odds of newborn chatbot reach than when the newborn’s birthing individual was White. Patients with public insurance (OR 0.63, 95% CI 0.55-0.71), self-pay (OR 0.56, 95% CI 0.38-0.83), and unknown insurance (OR 0.58, 95% CI 0.48-0.69) had lower odds of reach than patients with private insurance. Patients who received prenatal care at clinics external to the hospital, including Kaiser clinics (OR 0.30, 95% CI 0.26-0.35), other clinics (OR 0.50, 95% CI 0.44-0.57), unknown clinics (OR 0.40, 95% CI 0.32-0.48), had lower odds of postpartum chatbot reach compared to patients who received prenatal care within the hospital health system. Finally, caregivers of newborns who stayed in the hospital for 2‐4 days after birth (OR 1.21, 95% CI 1.09-1.35) had greater odds for chatbot reach compared to newborns who stayed in the hospital less than 2 days, but no significant differences were found for the newborns who stayed more than 4 days (OR 0.99, 95% CI 0.86-1.15).

**Table 4.** Multivariate logistic regression for newborn chatbot reach among caregivers of newborns discharged from the hospital between October 2, 2022, and January 15, 2025. Reach is defined as the number of people who opened the chatbot link out of those who received outreach messaging.

Characteristics	OR^a^ (95% CI)	*P* values	
Age (continuous)	1.02 (1.01-1.03)	<.001	
Race			
White (reference)	N/A^b^	N/A	
Black	0.61 (0.50-0.74)	<.001	
Other	0.73 (0.59-0.91)	.004	
Unknown	0.74 (0.60-0.91)	.005	
Insurance			
Private (reference)	N/A	N/A	
Public	0.63 (0.55-0.71)	<.001	
Self-pay	0.56 (0.38-0.83)	.004	
Other	0.85 (0.48-1.53)	.60	
Unknown	0.58 (0.48-0.69)	<.001	
Time in hospital			
<2 days (reference)	N/A	N/A	
2‐4 days	1.21 (1.09-1.35)	.001	
>4 days	0.99 (0.86-1.15)	.92	
Prenatal location			
Within the hospital integrated health system (reference)	N/A	N/A	
Kaiser clinics^c^	0.30 (0.26-0.35)	<.001	
Other external clinics (eg, FQHCs)^d^	0.50 (0.44-0.57)	<.001	
Unknown clinics	0.40 (0.32-0.48)	<.001	

a OR: odds ratio.

b N/A: not applicable.

c Kaiser clinics are external clinics where both care and insurance are provided within the same system.

d FQHC: federally qualified health center.

We conducted additional analyses limited to those where we had information on birthweight and gestational age (newborns discharged between October 2, 2022, and September 30, 2024). Birthing individual age, race, insurance, newborn time in the hospital, and prenatal care location remained in the model when including and excluding newborn birthweight and gestational age; ethnicity and preferred language were eliminated for both final models to fit the best model. Caregivers of newborns with very low birthweight (OR 2.44, 95% CI 1.32-4.50) had greater odds of opening the chatbot compared to newborns with normal birthweight, although no significant differences were found for the newborns with low birthweight (OR 1.11, 95% CI 0.88-1.39). In relation to gestational age, for newborns born at <37 weeks (OR 0.77, 95% CI 0.61-0.97), the caregivers were less likely to open the chatbot than caregivers with newborns born at term (37+ weeks). The ORs for other variables in the model were similar to those in the model with the full cohort that did not include birthweight and gestational age (Multimedia Appendix 2).

#### Chatbots Reach Changes Over Time

No significant changes in reach were found after launching the updated version of the newborn and postpartum outreach and chatbot content, nor after including the chatbot information in patients’ discharge paperwork (Table 5); thus, we focused on results in the combined analyses for the full time period. We found significant improvements in reach for Spanish-speaking patients after deploying the Spanish version of the newborn chatbot (OR 1.42, 95% CI 1.03-1.96), but no significant differences in reach were found for the postpartum chatbot (OR 1.09, 95% CI 0.78-1.53). For a graphic description of the evolution of postpartum and newborn chatbots’ reach over time (MultimediaAppendices 3  4).

**Table 5.** Change in chatbot reach before and after changes in outreach, content, and awareness efforts.

Implementation strategies	Postpartum chatbot					Newborn chatbot				
	Total received	Total reach^a^	Reach, %	OR^b^ (95% CI)		Total received	Total reach^a^	Reach, %	OR (95% CI)	
Outreach and content revisions^c^										
Original content (reference)	3554	2278	64.1	N/A^d^		4212	2775	65.9	N/A	
New content	2610	1639	62.8	0.95 (0.85-1.05)		2935	1914	65.2	0.97 (0.88-1.07)	
Content in Spanish^e^										
Pre-Spanish language (reference)	448	277	61.8	N/A		519	301	58	N/A	
Spanish language	213	136	63.9	1.09 (0.78-1.53)		237	157	66.2	1.42 (1.03-1.96)	
Discharge paperwork^f^										
Prior (reference)	5150	3274	63.6	N/A		5967	3950	66.2	N/A	
Included in discharge paperwork	1355	866	63.9	1.02 (0.90-1.15)		1522	983	64.6	0.93 (0.83-1.05)	

a Reach is defined as the number of people who opened the chatbot link out of those who received outreach messaging.

b OR: odds ratio.

c Individuals discharged before January 9, 2024, for the postpartum chatbot and January 13, 2024, for the newborn chatbot were included in the analysis as the original content group, and individuals discharged between February 20, 2024, and January 15, 2025, for both chatbots were included in the new content group.

d N/A: not applicable.

e Only Spanish-speaking individuals identified in the medical record were included in the analysis. Individuals discharged before April 21, 2024, for the postpartum chatbot and April 25, 2024, for the newborn chatbot were included in the analysis as the pre-Spanish language group, and patients discharged from June 2, 2024, through January 15, 2025, were included in the Spanish-language group.

f Individuals discharged before July 29, 2024, were included in the analysis as before discharge paperwork, and individuals discharged from July 29, 2024, through January 15, 2025, were included in the discharge paperwork group.

### Lessons Learned

This project highlighted the importance of involving stakeholders, including clinical staff, providers, patients, and community before and during the chatbot implementation to ensure acceptability, usefulness, and reach of the chatbot. The clinical teams were supportive of the rollout throughout the project. However, there were significant challenges in data collection and reporting from the third-party vendor, which may be better addressed in future work by working through specific needs, data accuracy, and various use cases for data before deploying the chatbot. Also, relying on a third-party vendor with ongoing expenses for hosting the chatbot limited the sustainability of this project after the project funding ended. Other health care systems or groups may consider options that are integrated into existing licenses so that ongoing fees are not prohibitive. Finally, given the design of the project and the vendor’s limited capability to track individual level engagement with the program over time, we were unable to assess the impact of the chatbot on health outcomes.

## Discussion

### Principal Findings

This study describes strategies to develop a postpartum and newborn chatbot that was offered as standard of care for birthing individuals and caregivers of newborns discharged at a large hospital that serves a socioeconomically and racially diverse population. The chatbots’ reach was close to two-thirds of the eligible birthing individuals and caregivers. Differences in reach were identified by age, race, prenatal care location, and insurance status for both chatbots, while newborn weight at birth, gestational age, and newborn time in the hospital were also significant predictors of the newborn chatbot reach. While disparities were present, this study demonstrated that chatbots have the potential to reach a significant proportion of this at-risk population and elucidates opportunities to conduct additional targeted supports.

Previous commercial and research programs have shown satisfaction among those who choose to receive messages about maternal and infant care. In other large-scale programs such as Text4Baby, a national SMS text messaging strategy in the US, about 150,000 individuals have chosen to enroll annually to receive SMS text messaging about prenatal, postpartum, and infant health recommendations, with users reporting high satisfaction [8]. Other interventions have also shown that patients and caregivers report high satisfaction when receiving SMS text messaging with health recommendations about postpartum depression [19] and infant care [7]. Health chatbots are also positively viewed [20,21], and parents tend to appreciate the informational and emotional support provided by chatbots [21]. In addition to high acceptability, mHealth strategies are also effective in improving health behaviors. For example, mHealth strategies have been shown to be effective in infant emergency room visit reduction [7], blood pressure monitoring [22], prenatal and postpartum weight management [23], and smoking cessation [24], among others [25].

In this project, about 65% of the individuals opened the chatbots, suggesting interest in reviewing information about postpartum and newborn care. In a randomized control trial, patients who received Rosie, an AI chatbot for new mothers, found that 87% (13/15) of the users reported using the chatbot at least weekly [13]. Another study of a postpartum AI chatbot found that of the 290 enrolled patients in the study, 99% of the patients responded to the platform at least once, and 52% asked a question to the chatbot [26]. However, to our knowledge, there are no comparable standard of care for newborn and postpartum mHealth educational outreach strategies that have evaluated reach in real-world settings, underscoring the need for this work. Prior real-world behavioral interventions on various health topics have documented SMS text messaging reach ranging from 14% to 60% [27-29]; our findings thus surpassed expectations. For example, a study among smokers and at-risk drinkers found that a total of 14% of the patients clicked on the embedded link to the apps in the SMS text messaging [27]. Another project using EHR data to identify smokers found that 15% responded to the smoking cessation SMS text messaging program after a single recruitment text [28]. Another project for patients aged 65 years and older found that 60% of the patients responded to the COVID-19 vaccine SMS text messaging outreach strategy [29].

We found notable differences in chatbot reach by age, race, insurance type, and prenatal care location for both chatbots. Lower chatbot reach among younger patients, Black patients, patients with public insurance, and those who received prenatal care at clinics external to the hospital health care system may be attributed to differences in desire for information, disparities in health/technological literacy, concerns about data plans, or trust in the health care system or chatbots [29,30]. We were also unable to deliver the chatbot to patients without a valid email or cell phone number in the EHR. To improve equity, it is crucial to develop interventions that address these issues, whether through additional in-clinic outreach to inform patients and caregivers about the chatbot, ensuring a valid email and phone number is documented in the EHR before discharge, or improving patient access to health information via different channels (eg, phone calls, SMS text messaging, and community outreach). Costs to the health care system and potential models for covering such low-cost services should also be explored. Future research could also consider intentional partnership with community groups focused on Black birthing individuals and underserved populations to avoid disparities in chatbot implementation.

While significant changes to the chatbot content and strategies were made to improve the chatbot’s reach, these strategies did not result in significant improvements. Similar findings were found in a prior study where no significant improvements in acceptability were found after intervention refinement based on participants’ feedback [30]. Our study suggests that further research is needed to understand the drivers of chatbot reach post-hospital discharge, such as user experience and how outreach and content can be optimized to capture and retain user interest over time. Additional strategies, such as identifying the best outreach time of day, further tailoring the outreach messages/content, and potentially including AI for personalization [13] may play a role in how users access and engage with similar chatbots. Community-based approaches may also be useful for addressing concerns about chatbots and identifying ways to increase the attractiveness of chatbots to patients who experience disparities. In addition, these changes could potentially impact other areas of user experience that we did not measure, like usability, satisfaction, and engagement. Ongoing audit and feedback may also prevent gaps in study metrics and provide timely information on how patients are using these types of mHealth interventions.

### Limitations and Strengths

In this study, patients did not have the opportunity to request that the chatbots be sent to their preferred contact information, which could have impacted reach. Furthermore, because our chatbot followed a rule-based system with fixed logic for user interaction, it did not allow patients to ask questions or review the information in their preferred order. While efforts were made to match the content to when patients might need this information, for some patients, the information presented in the chatbots might not have been relevant. Future integration of conversation AI is recommended to provide personalized responses; however, it needs to be done with caution to guarantee that patients are receiving accurate information.

Two-thirds of the birthing individuals and most newborns seek postpartum or newborn care, respectively, outside the system. Thus, we could not evaluate the impact on patient health outcomes. Even within our system, there was limited ability to link engagement with specific outcomes due to the variability in the ways that individuals could engage with the chatbot and view tailored information. Future longitudinal studies within an integrated health care system, through partnership across facilities or designed in a manner where relevant outcome data can be linked to specific aspects of chatbot engagement, are also needed to assess the impact of chatbot reach and engagement on maternal and infant health outcomes, such as postpartum depression, follow-up care, and visits to the emergency room. Further research is also needed to understand patterns of reach related to gestational age, birthweight, or duration of hospital stay.

Despite these limitations, this study has many strengths. Delivering this program at scale provides unique information on who might engage with this type of digital outreach. This chatbot was intended to reach all patients and especially those who might not have a regular care provider to help them establish appropriate care. Finally, to our knowledge, our study presents the first example about chatbot reach to birthing individuals and newborn caregivers as standard of care within an urban hospital.

### Conclusions

Our intervention demonstrated that chatbots can deliver important health information to many individuals efficiently, offering timely guidance on postpartum recovery, infant care, and follow-up appointments. These tools may serve as a supplement to patient care, helping bridge gaps in communication and support between health care providers and patients, especially for those who do not seek regular care. Understanding how constant interaction with digital health tools influences clinical outcomes, including a focus on racially and socioeconomically diverse populations, will help refine usability and effectiveness of these technologies.

## Supplementary material

10.2196/81844 Multimedia Appendix 1 Postpartum and newborn chatbot outreach message schedules.

10.2196/81844 Multimedia Appendix 2 Multivariate logistic regression for newborn chatbot reach including birthweight and gestational age variables for patients discharged from October 2, 2022, to September 30, 2024.

10.2196/81844 Multimedia Appendix 3 Postpartum chatbot reach by week of initial outreach.

10.2196/81844 Multimedia Appendix 4 Caregivers’ newborn chatbot reach by week of initial outreach.

## Abbreviations

EHR: electronic medical record
FQHC: federally qualified health center
iCHECK-DH: Guidelines and Checklist for the Reporting on Digital Health Implementations
IRB: Institutional Review Board
mHealth: mobile health
