# Supplementary figures and images for: Increasing Use of a Postpartum and Newborn Chatbot among Birthing Individuals and Caregivers: Development and Implementation Study
Source: JMIR Pediatr Parent. 2026 Jan 9;9:e81844. doi: 10.2196/81844 (PMC12788709; doi:10.2196/81844)

**Figure S1.**


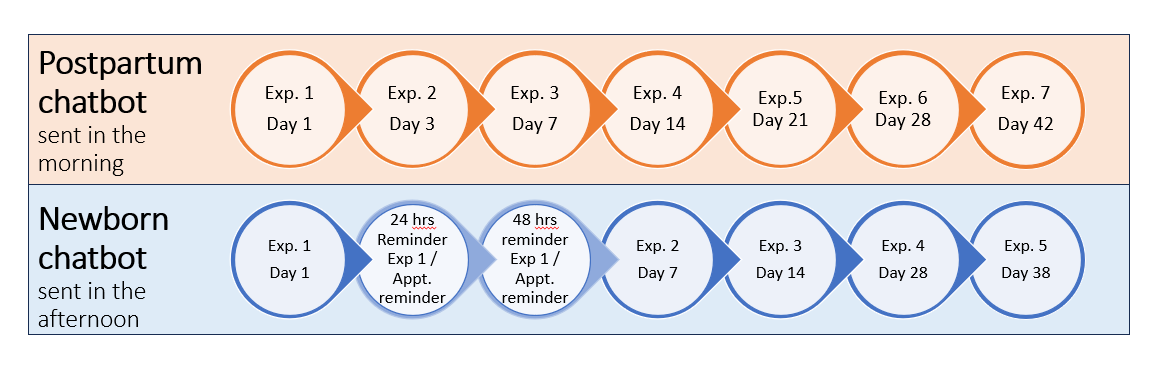


Exp. = Experience. Each chatbot experience provided unique content.

Supplement: Multimedia Appendix 1 [file pediatrics-v9-e81844-s001.docx]

**Figure S1.**


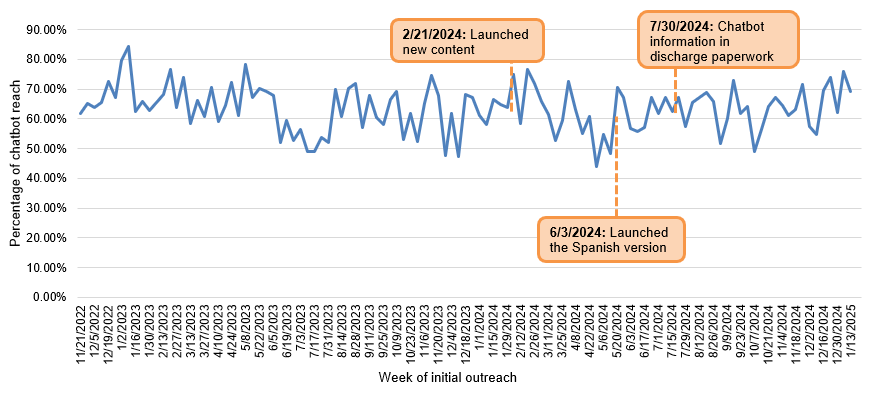

Supplement: Multimedia Appendix 3 [file pediatrics-v9-e81844-s003.docx]

**Figure S1.**


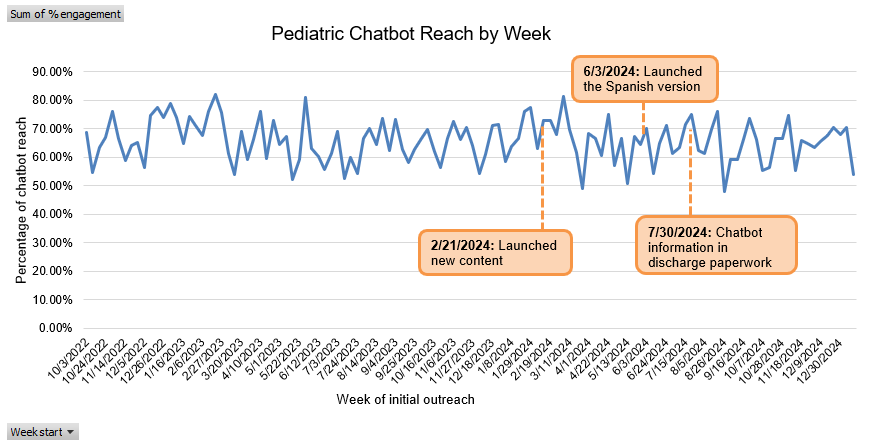

Supplement: Multimedia Appendix 4 [file pediatrics-v9-e81844-s004.docx]
