# Supplementary material for: Increasing Use of a Postpartum and Newborn Chatbot among Birthing Individuals and Caregivers: Development and Implementation Study
Source: JMIR Pediatr Parent. 2026 Jan 9;9:e81844. doi: 10.2196/81844 (PMC12788709; doi:10.2196/81844)
Supplement: Multimedia Appendix 2 [file pediatrics-v9-e81844-s002.docx]

**Table S1**.

|  | Odds Ratio | 95% CI^a^ | *p*-value |
| --- | --- | --- | --- |
| **Age (continuous)** | 1.02 | 1.01, 1.03 | *<.0001* |
| **Race** |  |  |  |
| White (ref.) |  |  |  |
| Black | 0.59 | 0.47, 0.73 | *<.0001* |
| Other | 0.70 | 0.55, 0.88 | *.003* |
| Unknown | 0.70 | 0.55, 0.88 | *.002* |
| **Insurance** |  |  |  |
| Private (ref.) |  |  |  |
| Public | 0.63 | 0.55, 0.72 | *<.0001* |
| Self-pay | 0.55 | 0.37, 0.84 | *.005* |
| Other | 0.90 | 0.47, 1.72 | *.75* |
| Unknown | 0.54 | 0.44, 0.65 | *<.0001* |
| **Time in hospital** |  |  |  |
| <2 days (ref.) |  |  |  |
| 2-4 days | 1.21 | 1.10, 1.38 | *<.001* |
| >4 days | 1.02 | 0.83, 1.25 | *.89* |
| **Newborn weight** |  |  |  |
| Very low birthweight (<1,500 g) | 2.44 | 1.32, 4.50 | *.004* |
| Low birthweight (1,500 to <2,500 g) | 1.11 | 0.88, 1.39 | *.38* |
| Normal birthweight (≥2,500 g; ref) |  |  |  |
| **Gestational age** |  |  |  |
| <37 weeks | 0.77 | 0.61, 0.97 | *.03* |
| ≥37 weeks (ref.) |  |  |  |
| **Prenatal location** |  |  |  |
| Within the hospital integrated health system (ref.) |  |  |  |
| Kaiser clinics^b^ | 0.29 | 0.25, 0.34 | *<.0001* |
| Other external clinics | 0.49 | 0.43, 0.56 | *<.0001* |
| Unknown clinics | 0.37 | 0.30, 0.46 | *<.0001* |

^a^ 95% Confidence Interval

^b^ Kaiser clinics are external clinics where both care and insurance are provided within the same system
